# Chinese Mobile Health Apps for Preventing and Managing Pelvic Floor Dysfunction: Quality Assessment and Content Analysis

**DOI:** 10.2196/80126

**Published:** 2026-06-24

**Authors:** Yuqing Song, Xue Deng, Ting Hu, Sijie Feng, Lu Xing

**Affiliations:** 1 Department of Pelvic Floor Medicine Center Nursing, West China Second University Hospital, Sichuan University, No. 20, Section 3, South Renmin Road, Sichuan Province, Chengdu, 610041, China, 86 19827473462; 2 Key Laboratory of Birth Defects and Related Diseases of Women and Children (Sichuan University), Ministry of Education, Chengdu, Sichuan, China; 3 Department of Gynecology, Guangdong Women and Children Hospital, Guangzhou, China; 4 Department of Gynaecology and Obstetrics, West China Second University Hospital, Sichuan University, Chengdu, Sichuan, China

**Keywords:** pelvic floor dysfunction, app, mobile health, mHealth, pelvic floor, quality assessment

## Abstract

**Background:**

Pelvic floor dysfunction (PFD) is a highly prevalent health problem, encompassing urinary incontinence, emptying disorders of the bladder, fecal incontinence, emptying disorders of the bowel, pelvic organ prolapse, sexual dysfunction, and chronic pelvic pain. Mobile health (mHealth) interventions delivered through apps can provide remote health services to improve patient compliance and enhance treatment effectiveness. Although apps for preventing and managing PFD have been developed and used, the features and quality of these apps in China have not been systematically examined.

**Objective:**

This study aimed to systematically summarize the functions and evaluate the quality of the existing mHealth apps for preventing and managing all kinds of PFD, such as urinary incontinence, fecal incontinence, and chronic pelvic pain.

**Methods:**

We systematically searched for potential PFD apps on the Apple App Store, Huawei AppGallery, and VIVO App Store. Apps were included if they were free, designed for preventing or managing PFD, in the Chinese language, could be downloaded and run on Android, Harmony, or iOS operating systems (OS), and incorporated elements of preventing and managing PFD. We excluded apps that were intended for use by health care providers and not relevant to PFD. Apps that met the inclusion criteria were downloaded and included for final analysis. The user version of the Mobile App Rating Scale (uMARS) was used to assess the apps’ quality and summarize the apps’ functionality according to guidelines.

**Results:**

Of the 3897 apps screened, 46 apps that met the inclusion criteria were included in the final analysis. All apps were developed by corporations. More than half of the apps had download counts exceeding 10,000, and 24 (52.2%) apps scored 4 or higher in app stores. Furthermore, nearly half of the apps (n=21, 45.7%) had been updated within the past month at the time of retrieval. The overall uMARS scores ranged from 2.29 to 4.50, with a mean uMARS score of 3.46 (SD 0.50), which is considered acceptable quality. Based on uMARS scores, 15.2% (n=7) were rated as poor quality, 65.2% (n=30) as acceptable, and 19.6% (n=9) as good quality. More than half of the apps provided the functions of exercise (n=44, 95.7%), personal information recording (n=31, 67.4%), and health education (n=28, 60.9%). Only 5 apps provided 5 or more functions.

**Conclusions:**

The apps for PFD revealed acceptable quality, and the majority provided exercise, personal information recording, and health education functions. However, many apps lacked comprehensive functionalities and did not provide immediate feedback or high-quality educational information. Health care providers should follow international guidelines to create high-quality, evidence-based, multifunctional apps for PFD. Future studies should explore the effects of the apps and real-world user feedback data in clinical settings.

## Introduction

Pelvic floor dysfunction (PFD) refers to functional impairments of the musculature that support the bladder, bowel, and vagina, including urinary incontinence, fecal incontinence, pelvic organ prolapse, sexual dysfunction, and chronic pelvic pain [1,2]. PFD is a highly prevalent health problem that seriously affects the daily function, sexual function, self-image, and quality of life of patients [3-5]. In China, the prevalence of symptomatic pelvic organ prolapse is 9.6% [6], and the weighted prevalence of female urinary incontinence is 16.0% [7]. By comparison, in a study of 1446 women in Spain, urinary incontinence occurred in 55.8% (807) of the women, fecal incontinence in 10.4% (150), and symptomatic uterine prolapse in 14.0% (203) [8]. Patients with PFD require long-term care to manage their symptoms. However, only 10.7% of patients seek medical care [9].

Patients with PFD face challenges in accessing effective and high-quality health services, such as low patient motivation and frequent health care visits [10,11]. Mobile health (mHealth) interventions delivered through apps can overcome these challenges by offering convenient tools, reminders, and instructions for pelvic floor muscle training (PFMT), improving patient compliance and treatment effectiveness through remote monitoring and feedback [12,13]. A longitudinal study involving 3051 postmenopausal women with PFD demonstrated that PFMT combined with education delivered through a mobile app significantly reduced the impact of pelvic floor symptoms on daily life [14]. A systematic review [15] revealed that mHealth app–based PFMT could improve symptom severity, quality of life, and adherence to treatment among women with stress urinary incontinence or stress-predominant mixed urinary incontinence.

Many current mHealth apps for PFD mainly focus on delivering PFMT [15-18]. mHealth app quality, such as usability, accuracy of information, and functionality, affects the user experience, determines whether users continue to use the app, and raises concerns regarding their long-term effectiveness and reliability [19]. Systematic reviews [16-18] have highlighted several limitations in the current PFD-related mHealth apps. Many apps lack a solid evidence base, with insufficient involvement from health care professionals in their design and content validation [17,18]. Sudol et al [18] evaluated 23 English apps in female pelvic medicine and reconstructive surgery but did not summarize the functions of these apps. Kasoff et al [17] reviewed 83 English apps for PFD, with functions such as voiding and bowel diaries, symptom tracking, medication tracking, social function, reminder systems, and disease information. However, no published articles summarize publicly available Chinese apps for PFD.

It is also worth highlighting that health care professionals should identify high-quality apps for individuals. Standardized instruments, such as the Mobile App Rating Scale (MARS), can systematically assess the quality of mHealth apps [20]. The MARS has demonstrated high interrater reliability for evaluating mHealth app quality across diverse domains [20]. However, its administration requires specialized training and expertise in mHealth and the relevant health field [21]. The user version of MARS (uMARS) is a reliable and user-friendly measure of app quality that can be administered by target users without professional training in mHealth [21]. As mHealth apps are primarily accessed by end-users rather than professionals, the uMARS could identify high-quality and useful apps from users’ perspectives.

Although numerous mHealth apps have been developed and implemented for PFD, Chinese apps have not been systematically summarized or assessed. Thus, this study aimed to summarize the content and functions of mHealth apps in China for PFD and evaluate their quality using uMARS.

## Methods

### Study Purpose

This study aimed to systematically review, evaluate the quality, and summarize the content and function of the existing publicly available mHealth apps in China for preventing and managing PFD, including urinary incontinence, pelvic organ prolapse, fecal incontinence, emptying disorders of the bladder, emptying disorders of the bowel, sexual dysfunction, and chronic pelvic pain. This study was conducted following the PRISMA (Preferred Reporting Items for Systematic Reviews and Meta-Analyses) guidelines for systematic reviews [22].

### Search Strategy

We systematically searched mHealth apps for PFD between March 24 and 28, 2025. Android, Harmony, and iOS are the most popular smartphone operating systems in China [23]. Huawei AppGallery is the largest app store, though some relevant apps are not available on it [24]. Xiaomi Market, OPPO Software Store, and VIVO App Store are also among the top 5 app stores in China [24]. Thus, we searched apps in the 3 major Chinese app stores: Apple App Store, Huawei AppGallery, and VIVO App Store. We conducted preliminary searches and group discussions to determine the optimal keywords for the 3 app stores, ensuring that all relevant apps could be screened. We extracted keywords from existing reviews on PFD, used the keywords for an initial app search in app stores, and then revised the keywords. Subsequently, we conducted group discussions to supplement additional keywords and revise keywords that were irrelevant or unsuitable for the research topic. The final Chinese keywords included: pelvic floor (盆底), pelvic floor muscle (盆底肌), pelvic floor rehabilitation (盆底康复), pelvic floor muscle rehabilitation (盆底肌康复), pelvic floor dysfunction (盆底功能障碍), pelvic floor disorders (盆底功能障碍), pelvic floor training (盆底训练), pelvic floor exercise (盆底锻炼), pelvic floor muscle exercise (盆底肌锻炼), pelvic floor muscle training (盆底肌训练), pelvic floor functional exercise (盆底功能锻炼), Kegel exercises (凯格尔), pelvic floor management (盆底管理), pelvic floor muscle management (盆底肌管理), pelvic floor treatment (盆底治疗), pelvic floor muscle treatment (盆底肌治疗), pelvic organ prolapse (盆腔脏器脱垂), prolapse (脱垂), uterine prolapse (子宫脱垂), vaginal prolapse (阴道脱垂), cystocele or bladder prolapse (膀胱脱垂), rectocele or rectal prolapse (直肠脱垂), urinary incontinence (尿失禁), overactive bladder (膀胱过度活动症), defecation dysfunction (排便功能障碍), fecal incontinence (大便失禁/粪失禁), and sexual dysfunction (性功能障碍). Two researchers used the above keywords to search apps in the 3 app stores and recorded all results.

### Inclusion and Exclusion Criteria

Apps were included if they met the following criteria: (1) designed for preventing or managing PFD; (2) available for free download; (3) in the Chinese language; (4) available for download from the Apple App Store, Huawei AppGallery, and VIVO App Store; and (5) able to run on Android, iOS, and Harmony operating systems. We reviewed the descriptions on app stores, downloaded and tested the apps, and excluded those not specifically focused on PFD (eg, general women’s health apps without a pelvic floor component) or those intended for use by health care providers.

### App Quality Assessment

We used the Chinese version of the uMARS to assess the apps’ quality [25]. uMARS is a feasible and reliable tool for evaluating mHealth apps, specifically designed for users without mHealth expertise [20,25]. As our team did not include a mHealth specialist, we selected uMARS as the appropriate assessment tool. The uMARS includes 14 items divided into 3 subscales: information, functionality, and engagement. All items were rated on a 5-point Likert scale from 1 (inadequate) to 5 (excellent). Mean scores were calculated for each subscale, and an overall mean score was calculated across all 3 subscales. Apps scoring ≥3 out of 5 on the uMARS were considered to be of acceptable quality, whereas those scoring ≥4 were rated as high quality.

In the quality assessment stage, 2 researchers independently assessed the apps’ quality. Initially, the researchers were required to thoroughly review the original uMARS development and translation literature to ensure a comprehensive understanding of the uMARS content. Then, the researchers independently evaluated other apps to establish consistent uMARS scoring rules. If the researchers’ initial ratings differed by 2 or more points, they discussed the differences to reach a consensus. If they couldn’t agree, a third researcher was brought in to resolve the issues. Finally, they downloaded the included apps and used them for at least 10 minutes to explore core features, the user interface, and navigational features before completing the uMARS evaluation. Pilot testing confirmed that 10 minutes was sufficient for a comprehensive app evaluation, so the researchers established 10 minutes as the evaluation threshold.

### Data Extraction

Descriptive data were manually extracted by 2 independent researchers after reading the initial descriptions and purposes of each mHealth app on the app stores. The extracted information included general details and functionalities.

1. App Information: app name, app store, platform, developer, download count, update time, star rating, and privacy policy. The definitions of app information are presented in Table 1.
2. Function: We summarized the strategies for preventing and managing PFD from guidelines [1,26]. The functions of PFD apps were categorized into personal information recording, exercise, physical therapy, health education, self-assessment, online community, and reminder systems.

**Table 1.** General information collected for each app.

Assessment measure	Definition
App store	Apps downloaded from the Apple App Store, Huawei AppGallery, and VIVO App Store.
Platform	Apps could operate on Harmony, Android, and iOS operating systems.
Developer	Apps were developed by a corporation or individual developer.
Download count	Number of apps downloaded in Huawei AppGallery and VIVO App Store. If the number of app downloads in Huawei AppGallery and VIVO App Store was different, we recorded the higher number.
Update time	The latest update time when we retrieved apps.
Star rating	Star rating (out of 5) left by users in the app store. If the star rating was different in the 3 app stores, we recorded the rating that had the highest number of evaluations.

Personal information recording includes collecting demographic data, obstetric history, disease-related data, and symptom details. The function of exercise focuses on pelvic floor muscle exercise guidance, including audio instructions, visual guides, customizable plans, and muscle contraction and relaxation tracking. As for the physical therapy function, apps linked with biofeedback devices can detect pelvic floor muscle activity, provide real-time visual feedback for correct activation, and integrate electrical stimulation. The health education function provides educational information about PFD, including general information about PFD, prevention strategies, treatment options, exercise guidance, and lifestyle modifications. Self-assessment enables users to evaluate and track pelvic floor symptoms using validated questionnaires, wearable devices, and bladder and bowel diaries. The online community enables individuals with PFD to exchange knowledge, offer emotional support, and boost collective self-efficacy through digital communication. Reminder systems include medication reminders, voiding reminders, fluid intake reminders, and exercise reminders with customizable timing.

### Data Synthesis

We used Excel (Microsoft Corp) to extract the apps’ general information and functionality and to record the scores of uMARS. SPSS 27.0 (IBM Corp) was used for statistical analysis. The quantitative variables are described by the mean and SD. The count data were presented as frequencies and percentages. The uMARS and subscale scores were calculated as the mean scores of the ratings provided by the 2 researchers. We used intraclass correlation coefficients to analyze the consistency of the 2 researchers at the subscale and overall score levels.

## Results

### App Selection

**Figure 1.**
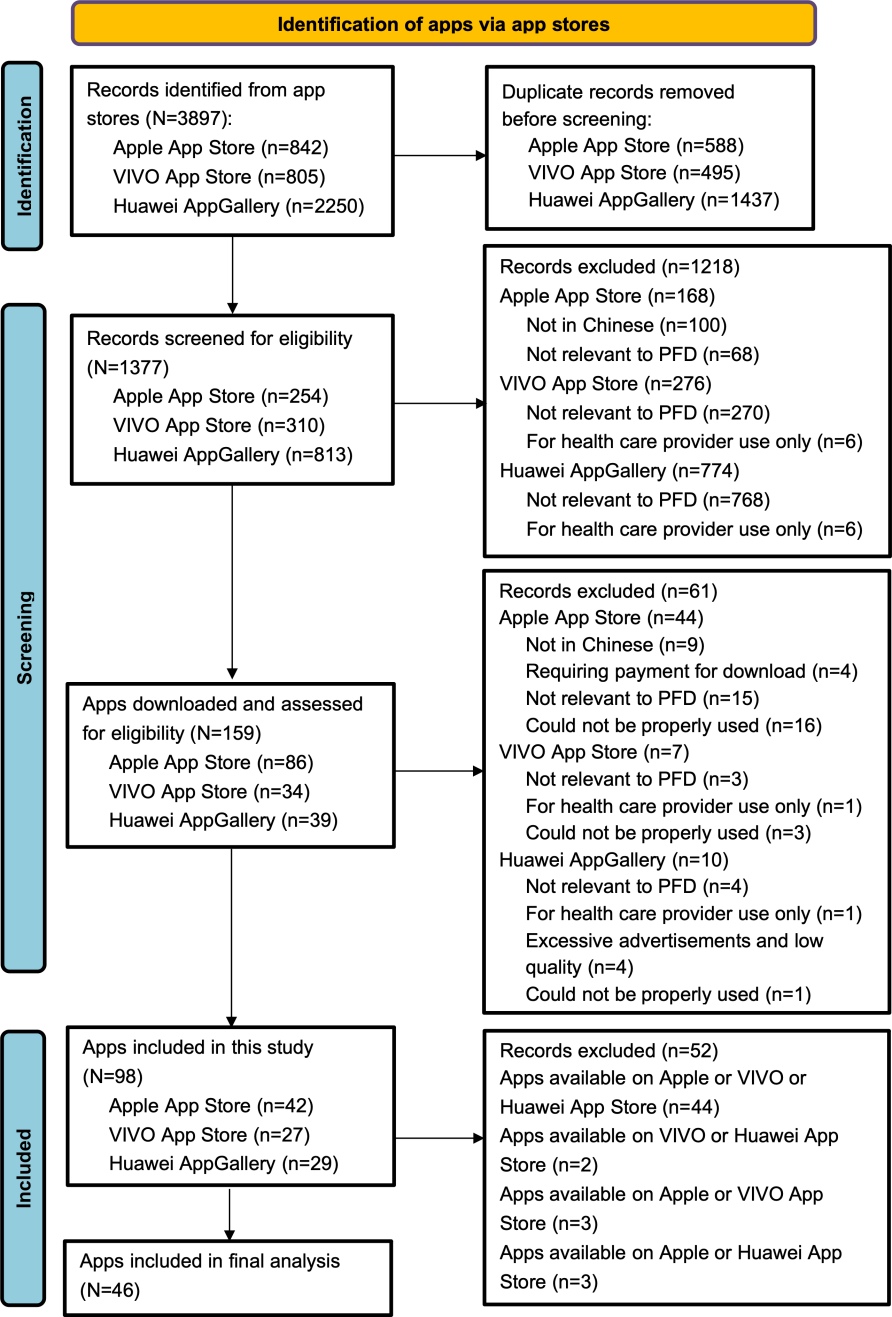
Flowchart of the selection of apps. PFD: pelvic floor dysfunction.

Our initial search identified 3897 apps, including 842 apps from the Apple App Store, 805 apps from the VIVO App Store, and 2250 apps from the Huawei AppGallery. After the elimination of duplicate apps, the final count comprised 310 Android apps, 813 HarmonyOS apps, and 254 iOS apps. After an initial screening based on the inclusion and exclusion criteria, we downloaded and analyzed 86 iOS apps, 34 Android apps, and 39 HarmonyOS apps. Subsequently, 61 apps were excluded: 9 were not in English, 20 were either technically inaccessible or required external device connectivity (eg, vaginal pelvic floor therapy devices) for use, 22 were irrelevant to the research topic, 4 required payment for download, 2 targeted health care professionals, and 4 had excessive advertisements and poor quality. Finally, 42 iOS apps, 27 Android apps, and 29 Harmony operating system apps were included in this study. Of the 98 included apps, 22 apps were available on all 3 app stores (counted as 3 apps), and 8 apps were available on 2 app stores (counted as 2 apps). After excluding 52 redundantly counted apps, we included 46 apps in this study (Figure 1).

### General Characteristics of the Apps

More than half of the apps (19/32, 59.4%) had download counts exceeding 10,000, and 24/46 (52.2%) apps scored 4 or higher. Nearly half of the apps (21/46, 45.7%) had recent updates (<1 mo), while 14/46 (30.4%) apps were updated between 1 month and 1 year, and 11/46 (23.9%) apps were outdated (>1 y) (Table 2).

**Table 2.** Characteristics of the apps for preventing and managing pelvic floor dysfunction (PFD; N=46).

Characteristic	Apps, n (%)
App store	
Apple App Store	42 (91.3)
Huawei AppGallery	29 (63.0)
VIVO App Store	27 (58.7)
Platform	
Harmony and Android and iOS	22 (47.8)
Harmony and Android	2 (4.3)
Harmony and iOS	3 (6.5)
Android and iOS	3 (6.5)
Harmony	2 (4.3)
iOS	14 (30.4)
Developer	
Individual developer	0 (0)
Corporation	46 (100)
Download count^a^ (n=32)	
0‐9999	13 (40.6)
10,000‐99,999	4 (12.5)
100,000‐999,999	7 (21.9)
1,000,000‐9,999,999	6 (18.8)
≥10,000,000	2 (6.3)
Update time	
>1 year	11 (23.9)
>1 month and <1 year	14 (30.4)
<1 month	21 (45.7)
Star rating	
0‐2.9	3 (6.5)
3.0‐3.9	7 (15.3)
4.0‐5.0	24 (52.2)
No rating	12 (26.1)

a Download counts were available only for Android and HarmonyOS apps, and 32 apps reported download data.

### Evaluation of the Included Apps

The intraclass correlation coefficient of the uMARS score was 0.970 (95% CI 0.946‐0.983). Three subscales demonstrated high consistency, with intraclass correlation coefficients as follows: information, 0.942 (95% CI 0.896‐0.968); function, 0.839 (95% CI 0.709‐0.911); engagement, 0.971 (95% CI 0.947‐0.984).

The mean total score of uMARS was 3.46 (SD 0.50), ranging from 2.29 to 4.50. According to the uMARS scores, 7 (15.2%) were classified as poor quality, 30 (65.2%) were categorized as acceptable quality, and 9 (19.6%) were designated as good quality. The mean scores for functionality, information, and engagement were 3.67 (SD 0.51), 3.56 (SD 0.45), and 2.96 (SD 0.83), respectively. The results are shown in Table 3 and Multimedia Appendix 1.

**Table 3.** App quality measured by uMARS^a^.

Variables	Values	Min-Max	
Overall uMARS, mean (SD)	3.46 (0.50)	2.29-4.50	
App quality stratification, n (%)			
Excellent (5)	0 (0)	—^b^	
Good (4≤5)	9 (19.6)	—	
Acceptable (3≤4)	30 (65.2)	—	
Poor (2≤3)	7 (15.2)	—	
Inadequate (1≤2)	0 (0)	—	
Information, mean (SD)	3.56 (0.45)	2.57-4.57	
Functionality, mean (SD)	3.67 (0.51)	2.50-4.75	
Engagement, mean (SD)	2.96 (0.83)	1.33-4.33	

a uMARS, the user version of the Mobile App Rating Scale.

b Not applicable.

### Functionality Summary of the Included Apps

More than half of the apps offered functionalities that included health education, exercise, and personal information recording (Table 4). In multifunctional apps, the most prevalent combination of functionalities included personal information recording, exercise, and health education, accounting for 21.8% (10/46) of the included apps. Only 5 out of 46 (10.9%) apps provided 5 to 6 functions.

**Table 4.** Functionality summary of the included apps.

Functionality	Values, n (%)
Reminder systems	6 (13.0)
Online community	9 (19.6)
Health education	28 (60.9)
Physical therapy	12 (26.1)
Exercise	44 (95.7)
Self-assessment	20 (43.5)
Personal information recording	31 (67.4)

#### Personal Information Recording

A total of 31 apps offered personal information recording. The apps enabled users to record their demographic data and disease-related data. Some apps could also record and track users’ exercise routines and pelvic floor symptoms.

#### Self-Assessment

Nearly half of the apps (20/46, 43.5%) offered self-assessment functionality that enabled users to evaluate their pelvic floor symptoms and health conditions. Many apps primarily assessed users’ basic information and performed pelvic floor muscle contraction and relaxation testing to identify users’ pelvic floor function and symptoms. Only a few apps provided validated instruments to help users assess their pelvic floor symptoms and other related health conditions.

#### Exercise

Almost all apps (44/46, 95.7%) offered PFMT (including audio or video-guided training) to various user groups: postpartum women, adolescent females, middle-aged and older women, and males. However, only 9 apps clearly stated that they were suitable for males. Users received instructions via audio or video to consistently and accurately contract and relax their pelvic floor muscles in synchrony with the accompanying music. Nineteen apps also offered paid courses guided by physicians or fitness instructors to help users learn and perform PFMT more effectively.

#### Physical Therapy

Only 12 apps offered a physical therapy function. To access the physical therapy functionality, these apps must be paired with a pelvic floor therapy device. Users should purchase a pelvic floor therapy device, connect it to the apps, and then perform pelvic muscle rehabilitation therapy at home.

#### Health Education

More than half of the apps (28/46, 60.9%) offered the function of pelvic floor health education. The educational content included articles and videos. Articles mainly explained what PFD is and how to perform PFMT, but much of the content was basic and lacked scientific rigor. Videos, which were primarily presented by health care professionals, offered accurate information on the prevention and management of PFD. However, only 6 apps included video-based educational content.

#### Online Community

The online communication function was present in 9 (19.6%) apps, offering a social platform for pelvic floor health. Within this community, health care providers and users could engage in communication through text and images. In most apps’ online communities, users primarily interacted to share their experiences regarding pelvic floor health, exercises, and daily life. Only 1 app offered health professional support, with experts addressing patients’ questions about PFD.

#### Reminder Systems

The functionality of reminder systems was offered in 6 apps. This functionality enabled users to set reminders for pelvic floor muscle exercises and menstrual cycle tracking. Additionally, this functionality facilitated the tracking of menstrual cycles, which could be beneficial for the overall women’s health management.

## Discussion

### Principal Results

This study systematically summarized the functions and evaluated the quality of 46 apps available in Chinese app stores that are designed for PFD. All included apps contained at least 1 of the 7 identified functions, with the majority providing functions related to health education, exercise, and personal information recording. Only 10.9% of the apps provided 5 or more of the identified functions. In this study, the mean uMARS score of the apps was 3.46, indicating acceptable quality. In other studies, the mean uMARS scores for eye-care apps, artificial intelligence–based apps, and mental health apps also ranged from 3.45 to 3.54 [19,27,28]. Kasoff et al [17] revealed that the quality scores of apps for PFD were low, and the most common functions were reminder systems, bowel diaries, exercise tracking, and voiding diaries. This suggests a considerable gap between guideline recommendations, the service capacity, and the quality of currently available PFD-related apps. We only reported descriptive uMARS scores and function counts, but did not evaluate clinical effectiveness. We suggest that the effectiveness of these apps should be explored in clinical trials and practice.

The overall quality of PFD-related apps was acceptable, which was similar to findings from other previous studies [17,27,28]. The results suggest that the current quality of apps remains suboptimal and warrants further enhancement. Across all assessed subscales, functionality achieved the highest average score, followed by information, while engagement received the lowest score. Previous studies [29-31] of apps targeting breast cancer, medicine, and medication management also reported the lowest scores in engagement and the highest scores in functionality. App developers have prioritized functionality, leading to notable improvements in app quality and usability in recent years [32]. Since mHealth apps primarily target doctors or patients, it is difficult to make users feel interested and relaxed when facing diseases [29]. This may contribute to the lowest scores in engagement [29]. This study used descriptive uMARS scores to rate the quality of apps. However, user engagement and participation, as well as the long-term effects of the apps on health outcomes, are also crucial for evaluating apps’ quality. Thus, health care professionals should explore the sustained use and effects of apps in future studies and clinical settings.

PFMT, such as Kegel exercises, is widely recommended for preventing and managing PFD [1]. Numerous studies [15,33,34] have revealed that PFMT delivered through mHealth apps is feasible and effective in improving health outcomes and exercise adherence. This study also found that almost all PFD-related apps (44/46, 95.7%) offered the feature of PFMT, which is consistent with findings from other studies [14,17,35]. Many apps in our study provide step-by-step voice instructions and video demonstrations to guide users through these exercises. Previous studies [14,36,37] have also reported that many apps provided simple instructions to show patients images, videos, or sounds indicating when to contract and relax pelvic floor muscles. Immediate feedback can enhance user engagement by allowing users to monitor their improvement over time [38]. However, most apps lack personalized feedback and professional guidance, which may limit the effectiveness of these exercises.

More than half of the PFD-related apps (31/46) offered the function of recording personal information. However, these apps merely provided basic exercise information logging and generated charts to visualize exercise conditions. Literature [39,40] suggests that the patient health record function should shift from simple data access to data modification, automated assessment, prediction, and recommendation. In this study, 43.5% of the apps provided a self-assessment function. Most apps guided patients through pelvic floor muscle contractions and relaxations to assess their pelvic floor muscle function, but only a few apps offered validated instruments to assess and track users’ symptoms. One study [41] also reported that few apps used validated instruments or wearable devices to enable users to conduct self-assessments.

The guidelines and reviews [1,42] have emphasized the importance of patient education for PFD. In this study, the educational function was offered in 60.9% of the PFD-related apps. The educational information consisted primarily of low-quality articles, which may not be tailored to users’ needs and preferences [43]. Liu et al [19] found that eye-care education delivered through video format was more engaging than text-based education, due to the dynamic audio-visual elements of the videos. Byambasuren et al [44] summarized that educational information on mHealth apps often consisted of low-quality educational articles. In a review of apps for pelvic organ prolapse and urinary incontinence [16], 67.9% of apps incorporated an educational or informational component, but only 17.9% of apps clearly delineated the sources for their information. Thus, future apps should incorporate high-quality, evidence-based educational information.

Some apps incorporated the functionalities of physical therapy, online community, and reminder systems. Pelvic floor physical therapy is the primary treatment for PFD [45,46]. Apps with physical therapy functions should be connected with pelvic floor therapy devices. However, the effects of physical therapy in apps remain unknown. Online communication enabled users and health care providers to share health information, emotions, and experiences [47]. In this study, only 1 app enabled communication between health care providers and users. We found that the reminder feature was offered in 6 apps. The reminder function of mHealth apps could improve users’ adherence to treatment [48]. Future apps should incorporate a variety of functionalities to achieve the prevention and management of PFD.

### Limitations and Future Work

This study systematically searched for and analyzed the publicly available mHealth apps in 3 different app stores. Meanwhile, we used a validated instrument, uMARS, to measure app quality. However, there were several limitations in this study. First, we only included apps in app stores that were freely accessible to the public. Paid apps were excluded due to a lack of funding. Second, we did not include the apps reported in the published literature, as many of these apps could not be downloaded or executed from the app stores. This could lead to selection bias, as apps reported in the literature may have higher quality and stronger evidence of effectiveness. Third, we only included Chinese-language apps due to limited funding and human resources. Thus, future studies should systematically summarize Chinese and other language apps from app stores and literature. Fourth, an important limitation was that we only provided descriptive data on uMARS scores and functionality counts. Health care professionals should explore the actual effects of apps and report real-world user feedback data in clinical settings. Finally, this study was limited in that the evaluation of apps relied on self-reported data by developers (eg, app descriptions, download counts, and self-rating scores) or users (eg, uMARS scores).

### Conclusions

This study analyzed the content and quality of 46 PFD-related apps in 3 Chinese app stores. The PFD-related apps demonstrated acceptable quality, and the majority provided functions such as health education, exercise, and personal information recording. However, many apps lacked comprehensive functions for PFD prevention and management, as well as immediate feedback and high-quality educational information. Health care providers are encouraged to adhere to international guidelines to develop high-quality, evidence-based multifunctional apps for the prevention and management of PFD. Future studies should investigate the effects of these apps and incorporate real-world user feedback data in clinical settings.

## Supplementary material

10.2196/80126 Multimedia Appendix 1 Mobile App Rating Scale scoring of the included apps.

## Abbreviations

mHealth: mobile health
PFD: pelvic floor dysfunction
PFMT: pelvic floor muscle training
PRISMA: Preferred Reporting Items for Systematic Reviews and Meta-Analyses
uMARS: the user version of the Mobile App Rating Scale
