# Supplementary material for: Chinese Mobile Health Apps for Preventing and Managing Pelvic Floor Dysfunction: Quality Assessment and Content Analysis
Source: J Med Internet Res. 2026 Jun 24;28:e80126. doi: 10.2196/80126 (PMC13293565; doi:10.2196/80126)
Supplement: Multimedia Appendix 1 [file jmir-v28-e80126-s001.docx]

**Multimedia Appendix 1 Mobile App Rating Scale scoring of included apps**

| Number | Name of apps | Information | Functionality | Engagement | The overall uMARS score |
| --- | --- | --- | --- | --- | --- |
| 1 | 7动凯格尔成人运动 | 4.29 | 4.00 | 4.33 | 4.21 |
| 2 | 9动PC肌凯格尔运动 | 3.14 | 3.25 | 2.00 | 2.93 |
| 3 | G动 | 3.86 | 4.25 | 3.33 | 3.86 |
| 4 | in动 | 3.57 | 2.50 | 2.00 | 2.93 |
| 5 | M8智能盆底 | 3.43 | 3.75 | 2.67 | 3.36 |
| 6 | 大悦盆底肌 | 3.86 | 3.50 | 2.67 | 3.50 |
| 7 | 和睿盆底康复 | 4.00 | 4.13 | 4.00 | 4.04 |
| 8 | 凯格尔 | 2.57 | 2.50 | 1.33 | 2.29 |
| 9 | 凯格尔Go | 3.43 | 3.75 | 4.00 | 3.64 |
| 10 | 凯格尔成人PC运动 | 3.57 | 4.00 | 2.33 | 3.43 |
| 11 | 凯格尔大师 | 3.43 | 3.75 | 3.67 | 3.57 |
| 12 | 凯格尔训练 | 3.86 | 4.25 | 2.67 | 3.71 |
| 13 | 凯格尔运动 | 4.57 | 4.50 | 4.00 | 4.43 |
| 14 | 凯格尔运动pro | 3.71 | 4.00 | 2.67 | 3.57 |
| 15 | 澜渟 | 4.57 | 4.75 | 4.00 | 4.50 |
| 16 | 澜渟盆底 | 4.29 | 4.75 | 4.00 | 4.36 |
| 17 | 乐动凯格尔 | 3.71 | 3.25 | 3.67 | 3.57 |
| 18 | 乐普康复 | 3.29 | 3.00 | 2.33 | 3.00 |
| 19 | 么么康 | 3.43 | 3.25 | 1.67 | 3.00 |
| 20 | 每日美愈 | 4.00 | 4.00 | 4.33 | 4.07 |
| 21 | 盆底动力 | 3.57 | 3.50 | 3.67 | 3.57 |
| 22 | 盆友 | 3.14 | 3.50 | 3.33 | 3.29 |
| 23 | 昕佳康 | 3.21 | 4.00 | 2.67 | 3.32 |
| 24 | 云天开盆底 | 3.00 | 3.25 | 1.67 | 2.79 |
| 25 | 左点盆底肌 | 2.86 | 3.25 | 1.67 | 2.71 |
| 26 | 邦妮康复-守护您的盆底健康 | 3.43 | 3.75 | 3.67 | 3.57 |
| 27 | 久动 | 3.71 | 3.75 | 2.67 | 3.50 |
| 28 | 识黛 | 3.71 | 3.75 | 3.00 | 3.57 |
| 29 | 雅致健康-专业家用产后修复品牌 | 3.71 | 4.00 | 3.00 | 3.64 |
| 30 | Emy-盆底练习 | 3.43 | 3.75 | 1.67 | 3.14 |
| 31 | K动-凯格尔运动&pc肌、盆底肌锻炼 | 3.86 | 4.00 | 4.33 | 4.00 |
| 32 | PCJ训练 | 3.43 | 3.75 | 3.33 | 3.50 |
| 33 | T-love | 3.43 | 3.75 | 3.00 | 3.43 |
| 34 | 凯格尔达人-骨盆底运动 | 3.71 | 3.75 | 3.00 | 3.57 |
| 35 | 凯格尔运动 盆底肌锻炼 | 4.14 | 4.00 | 4.33 | 4.14 |
| 36 | 凯格尔运动 | 3.29 | 3.50 | 2.67 | 3.21 |
| 37 | 凯格尔运动男性 盆底肌训练Kegel Men | 3.29 | 3.50 | 3.50 | 3.39 |
| 38 | 凯格尔运动-盆底肌恢复练习 | 3.14 | 3.50 | 2.67 | 3.14 |
| 39 | 凯格尔运动-盆底肌训练凯格尔运动助手 | 2.71 | 3.25 | 2.67 | 2.86 |
| 40 | 凯格乐-智能盆底肌运动健康软件 | 3.29 | 3.25 | 2.33 | 3.07 |
| 41 | 每日凯格尔 | 3.14 | 3.25 | 2.67 | 3.07 |
| 42 | 每日提提 | 3.64 | 3.25 | 3.33 | 3.46 |
| 43 | 盆底生物刺激反馈仪软件 | 4.29 | 4.50 | 3.67 | 4.21 |
| 44 | 小欧健康 | 3.71 | 3.75 | 2.33 | 3.43 |
| 45 | 羽合盆底 | 3.43 | 3.25 | 2.67 | 3.21 |
| 46 | 智慧家康 | 3.00 | 2.75 | 1.67 | 2.64 |
|  |  |  |  |  |  |
